# Exercise as Medicine in Cardio-Oncology: Reducing Health Disparities in Hispanic and Latina Breast Cancer Survivors

**DOI:** 10.1007/s11912-023-01446-w

**Published:** 2023-09-16

**Authors:** Paola Gonzalo-Encabo, Nathalie Sami, Rebekah L. Wilson, Dong-Woo Kang, Salvatore Ficarra, Christina M. Dieli-Conwright

**Affiliations:** 1 Division of Population Sciences, Department of Medical Oncology, Dana-Farber Cancer Institute, 375 Longwood Avenue, Boston, MA 02215 USA; 2 Harvard Medical School, Boston, MA USA; 3 Universidad de Alcalá, Facultad de Medicina y Ciencias de la Salud, Departamento de Ciencias Biomédicas, Área de Educación Física y Deportiva, Madrid, Spain; 4 Department of Internal Medicine, Los Angeles County-University of Southern California Medical Center, Keck School of Medicine, Los Angeles, CA USA; 5 Sport and Exercise Sciences Research Unit, Department of Psychology, Educational Science and Human Movement, University of Palermo, Via Giovanni Pascoli 6, 90144 Palermo, Italy

**Keywords:** Cardio-oncology, Exercise, Physical Activity, Breast Cancer, Health Disparities

## Abstract

**Purpose of Review:**

This review aims to access the current state of the evidence in exercise as medicine for cardio-oncology in Hispanic and Latina breast cancer survivors and to provide our preliminary data on the effects of supervised aerobic and resistance training on cardiovascular disease (CVD) risk in this population.

**Recent Findings:**

Breast cancer survivors have a higher risk of CVD; particularly Hispanic and Latina breast cancer survivors have a higher burden than their White counterparts. Exercise has been shown to reduce CVD risk in breast cancer survivors; however, evidence in Hispanic and Latina breast cancer survivors is scarce.

**Summary:**

Our review highlights a clear need for exercise oncology clinical trials in Hispanic and Latina breast cancer survivors targeting CVD risk factors. Moreover, our exploratory results highlight that 16 weeks of aerobic and resistance training may reduce the 10-year risk of developing CVD by 15% in Hispanic and Latina breast cancer survivors.

## Introduction

Cardio-oncology is a subspeciality that arose from the need of detecting, monitoring, and treating cardiovascular disease occurring as a side-effect of cancer treatments [1]. Particularly, breast cancer survivors have a double risk of cardiovascular disease-related mortality than women without cancer history due to breast cancer-related treatments (i.e., anthracycline chemotherapy) [2]. Furthermore, Hispanic and Latina women have a higher burden of breast cancer mortality and cardiovascular risk factors when compared to non-Hispanic and Latina White women [3•, 4]. In this regard, exercise is known as a non-pharmacological strategy to reduce cardiovascular risk and cardiovascular risk factors in the general population [5]. In the past decades, clinical trials have focused on exercise as an strategy to reduce cardiovascular risk factors among breast cancer survivors [6], whereas Hispanic and Latina women with breast cancer have been unrepresented in previous research [6].

Herein, we provide a review of the current evidence on exercise interventions in Hispanic and Latina women with breast cancer targeting cardiovascular-related outcomes. Furthermore, we provide preliminary data on the effects of an exercise intervention on cardiovascular risk in Hispanic and Latina breast cancer survivor.

## Cardiovascular Disease after Breast Cancer

As a prominent public health issue [7], cardiovascular disease is the leading cause of mortality in both men and women in the USA with approximately 48% of American adults projected to develop cardiovascular disease during their lifespan, with roughly one in every three deaths attributed to cardiovascular disease [8]. This is particularly concerning in the context of breast cancer survivors as a number of studies have shown they have a greater incidence of cardiovascular disease, including heart failure and cardiac arrhythmias, compared with the general population [9•, 10]. Specifically, a recent study among breast cancer survivors indicated that cardiovascular disease was the second leading cause of death [9•], and a greater than two times higher risk of cardiovascular disease-related mortality when compared to women without a history of cancer [2, 11].

Cardiovascular disease and breast cancer have shared risk factors including age, diet, family history, alcohol intake, obesity/overweight, physical activity, and tobacco use [12]. However, factors relevant to breast cancer treatments, such as chemotherapy, radiation therapy to the heart, and endocrine therapy, are known for their potential to induce cardiotoxicity and subsequent cardiovascular disease development [13]. These anti-cancer agents can directly damage myocardial cells through reactive oxygen species (ROS), interfere with the mitochondrial function, cause cardiomyocyte death by damaging DNA in cardiac cells, and lead to vascular endothelial dysfunction, through which mechanisms patients experience cardiotoxicity or long-term cardiovascular complications [14]. Furthermore, breast cancer treatments may indirectly impact cardiovascular health through metabolic dysregulation, which refers to disruptions in the normal metabolic processes within the body, including energy production, glucose metabolism, and lipid metabolism [15]. Breast cancer treatment can elicit various metabolic conditions associated with cardiovascular disease, such as insulin resistance, lipid abnormalities, and increased fat mass [16]. These conditions can lead to detrimental cardiovascular outcomes, including blood vessel damage, heightened inflammation, atherosclerosis, and impaired cardiac function [17]. Therefore, cardiovascular disease prevention and management strategies should be carefully considered and implemented in breast cancer patients and survivors to mitigate the potential impact of shared risk factors and the cardiotoxic effects of breast cancer treatments [12, 17].

## Racial and Ethnic Disparities in Cardio-Oncology in Hispanic and Latina Women with Breast Cancer

Despite the Hispanic and Latina/o population being the second largest racial and ethnic community in the USA, it is one of the most underserved populations facing numerous health disparities and inequities in access to care [18, 19]. Specifically, Hispanic and Latina women have a higher burden of breast cancer mortality, advanced cancer stage at diagnosis, and worse prognosis compared to non-Latina/Hispanic White women [4, 20•]. Furthermore, epidemiologic studies have shown striking racial and ethnic disparities in cardio-oncology with Hispanic and Latina breast cancer survivors facing a higher risk of cardiovascular risk factors, cardiovascular disease, and cardiovascular-related mortality compared with non-Hispanic/Latina White women [3, 21].

Regarding specific risk factors for cardiovascular disease, Hispanic and Latina breast cancer survivors have higher rates of obesity compared with non-Latina/Hispanic White women [22, 23]. Particularly, visceral obesity is highly prevalent with data showing approximately a 75% prevalence of central obesity in Hispanic population versus 58% in non-Hispanic White [24]. Central obesity is associated with several conditions such as diabetes, hypertension, dyslipidemia, and metabolic syndrome, increasing cardiovascular risk [25]. Particularly, in breast cancer survivors, diabetes has been significantly associated with mortality outcomes with stronger associations found among Hispanic breast cancer survivors (hazard ratio (HR):1.85 95% CI 1.11–3.09) compared with non-Hispanic White (HR: 1.33 95% CI 0.67–2.62) [26]. Furthermore, previous data from our laboratory showed a 95% prevalence of metabolic syndrome in Hispanic and Latina breast cancer survivors after completing chemotherapy [27]. Moreover, disparities in treatment options may also increase the risk of cardiovascular disease, with a higher risk of heart diseases due to chemotherapy and diseases of veins and lymphatics due to hormone therapy in Hispanic and Latina breast cancer survivors compared to non-Hispanic/Latina White women [3•]. Access to specialty care contributes to cardiovascular health disparities. Cardio-oncology has rapidly grown in the last years; however, specialists in this field are still very limited and centralized in large medical institutions. Therefore, patients may face unique barriers in access to this specific care with transportation and lack of insurance coverage being important aspects to consider [28•]. Inequities in the lack of access to healthy food options and physical activity are also important risk factors for cardiovascular disease [29]. The National Center for Health Statistics has shown a high prevalence of physical inactivity in Hispanic individuals with approximately 67% not meeting the 2008 Physical activity Guidelines for Americans [30]. Therefore, cardio-oncology disparities in Hispanic and Latina women with breast cancer are due to complex interactions of social determinants of health that include economic, geographical, and cultural factors that influence health outcomes and behavior [31–33].

## Exercise Cardio-Oncology Research Is Lacking in Hispanic and Latina Women with Breast Cancer

Clinical strategies have been implemented to counteract cardiovascular risk factors in breast cancer survivors including modifications in treatments, monitoring of symptoms, and pharmacological strategies (i.e., beta-blockers, angiotensin-converting enzyme (ACE)-inhibitors, etc.) [34]. While these treatments have shown clinical benefits in reducing cardiovascular risk, they also have side effects [35]. Thus, there is a need of developing non-pharmacological adjuvant therapies to reduce cardiovascular risk and cardiovascular risk factors in breast cancer survivors. In this regard, lifestyle interventions may be an important strategy to reduce cardiovascular risk factors in Hispanic and Latina breast cancer survivors.

Exercise is known as a non-pharmacological strategy to reduce cardiovascular risk factors in the general population and in other clinical populations [5]. Current guidelines for exercise in cancer survivors from the American College of Sports Medicine recommend 150 min a week of aerobic exercise and at least 2 days per week of resistance training [36]. Furthermore, guidelines from the American Society of Clinical Oncology recommend regular aerobic and resistance training during cancer treatments [37]. Despite these recommendations, research has shown that approximately 14% of cancer patients and survivors meet these guidelines [38, 39]. Particularly Hispanic and Latina women report lower levels of physical activity when compared to their non-Hispanic White counterparts [40]. In this regard, Hispanic and Latina breast cancer patients may face specific structural and personal barriers to engaging in physical activity and exercise oncology clinical trials [41]. Examples of barriers to participation in oncology clinical trials include lack of transportation, lack of awareness in research, interference with work and family responsibility, financial costs, time constraints due to having multiple jobs and caring for more than one generation of family members, etc. [42] Regarding specific barriers to physical activity, structural barriers include neighborhood and community walkability and safety, distance to physical activity facilities [43, 44], as well as personal barriers in addition to the above reported by Hispanic and Latina breast cancer survivors include lack of enjoyment, lack of exercise literacy, self-consciousness, and discouragement [45].

While several clinical trials have focused on the effects of exercise in reducing cardiovascular risk factors among breast cancer survivors [6], Hispanic and Latina women with breast cancer have been largely underrepresented in exercise interventions [46]. We have reviewed the existing literature in the field of exercise oncology in Hispanic and Latina women with breast cancer targeting cardiovascular-related outcomes (Table 1). The following search queries were applied on PubMed and Web of Science: ((breast neoplasms[MeSH Terms]) OR (breast cancer)) AND ((hispanic) OR (latino) OR (latina) OR (Hispanic or Latino[MeSH Terms])) AND ((exercise[MeSH Terms]) OR (exercise) OR (physical activity)); ALL=(“breast cancer” OR “breast neoplasm”); ALL=(“hispanic” OR “latino” OR “latina”) AND ALL=(“exercise” OR “physical activity”). A total of 444 records were identified (179—PubMed, 265—Web of Science). This review was conducted by two independent reviewers that screened the literature. Relevant reviews’ reference list was also screened to find eligible studies. Experimental studies assessing the effects of exercise interventions on cardiovascular-related outcomes among Hispanic and Latina breast cancer survivors were included. Cardiovascular-related outcomes measured in these studies include those that have been associated with cardiovascular risk among breast cancer patients, including metabolic and inflammatory biomarkers (i.e., cholesterol, insulin, glucose, etc.) [12, 47], body composition (i.e., body fat, body mass index (BMI), weight, etc.) [48], and cardiorespiratory fitness (i.e., VO_2max_ testing, 6-min walk test, etc.) [49, 50].

**Table 1:** Studies characteristics and results summary

Author-year	Design	Sample (*N*)	% of H/L	Treatment phase	Stage	Mean Age (yr)	Mean BMI (kg*m^−2^)	PA levels	Intervention type	Study length (weeks)	Freq. (n/w)	Intensity (%)	Results CVD-related outcomes
Greenlee et al. 2012	RCT	38	79	>6 months post-treatment	0–IIIa	50.7±68.9	33.2±5.9	Sedentary	COMB (circuit)	24	3	<60% – 70–75% of HRmax (AT)	No changes in cardiorespiratory fitness (VO_2_max). Significant weight loss in IG vs CG (no significant diff. by ethnicity). No changes in metabolic biomarkers (cholesterol, TGs, glucose, hsCRP, insulin, total ghrelin, adiponectin, IGF, HOMA-IR)
Hughes et al. 2008	Single arm	25	100	>6 months post-treatment	I–IV	50±8.4	n/a	n/a	COMB	10	n/a	n/a	Significant increase in cardiorespiratory fitness (VO_2_max)
Lee et al. 2019	RCT	30	73	On chemotherapy	I–III	46.9±9.8	31.0±7.5	<30 min of PA/week	HIIT	8	3	10%/90% PPO (60rpm)	IG increased in PPO and VO_2_max, while CG significantly decreased both PPO and VO_2_max. No time×group interaction.
Lee et al. 2021													Cardiorespiratory fitness (6MWT) significantly increased in IG vs CG.
Ortiz et al. 2021	RCT	89	100	>3 months post-treatment	I–IV	55.4±10	31±6.5	Not active (ACSM definition)	COMB	16	2	n/a	Cardiorespiratory fitness (6MWT) increased in IG, without significant differences between IG and CG.
Owens et al. 2009	Quasi-experimental	13	100	During and after chemotherapy	I–II	51.5#	n/a	Not exercising	COMB	24	3	n/a	No significant changes in weight, BMI, % body fat, and fasting glucose.

*ACSM* American College of Sports Medicine, *BMI* body mass index, *CG* control group, *COMB* combined aerobic and resistance exercise, *Freq.* frequency, *F-U* follow-up, *H/L* Hispanic/Latina, *HIIT* high intensity interval training, *HOMA-IR* homeostasis model assessment-estimated insulin resistance, *HRmax* maximum heart rate, IG intervention group, *IGF* insulin-like growth factor, *n/a* not available, *PA* physical activity, *PF* physical fitness, *PPO* peak power output, *RCT* randomized controlled trial, *VO*_*2*_*max* maximal oxygen consumption

#calculated based on age range.

After the search, a total of 444 records were identified with only 5 studies meeting our criteria (Table 1). On average, the studies included 90.4% of Hispanic and Latina breast cancer survivors, aged 50.9±24.3 years with a mean body mass index (BMI) of 31.7±6.6 kg/m^2^. Exercise interventions were composed mostly of combined aerobic and resistance exercise, three exercise sessions per week for a duration of 16 weeks. Briefly, results from these studies showed that there is scarce evidence on the effects of exercise in reducing cardiovascular risk factors in Hispanic and Latina breast cancer survivors. Only two previous studies reported metabolic biomarkers and showed no differences between the exercise group and the control group [51, 52]. Conflicting results have been shown regarding cardiorespiratory fitness with studies reporting no changes [52–54], while one study found improvements in cardiorespiratory fitness after an exercise intervention [55, 56]. Regarding body composition parameters there are also conflicting results with one study reporting reductions in body weight after an exercise intervention [52], while one study reported no changes in weight and body composition [51].

Therefore, our review highlights the lack of exercise oncology clinical trials in Hispanic and Latina breast cancer survivors with a special emphasis on cardiovascular risk factors. The included studies have used surrogate outcomes of cardiovascular disease, but to our knowledge, no previous study has included specific outcomes of cardiovascular disease risk in this population. In this regard, one widely employed method for assessing cardiovascular disease risk is the Framingham Risk Score (FRS), which combines six risk factors—age, high-density lipoprotein cholesterol (HDL), low-density lipoprotein cholesterol (LDL), systolic blood pressure, diabetes, and smoking status [57]. Thus, studies examining the effects of exercise on specific outcomes of cardiovascular disease risk in Hispanic and Latina women with breast cancer are lacking.

## Preliminary Evidence of the Effects of Exercise on Cardiovascular Risk in Hispanic and Latina Women with Breast Cancer

To fill this gap, we conducted an exploratory analysis to assess cardiovascular disease risk using the FRS in a subsample of patients from our previous clinical trial. We previously reported that aerobic and resistance exercise reduced the FRS (−9.5; 95% CI, −13.0 to −6.0) and the 10-year FRS-predicted risk of developing cardiovascular disease by 11% in racially/ethnically diverse breast cancer survivors (*N*=100) with overweight or obesity [58]. The high number of Hispanic and Latina women in our study allowed us to explore the effects of the exercise intervention on the FRS and the 10-year FRS-predicted risk of developing cardiovascular disease specifically focusing on this particular at-risk population of breast cancer survivors [59].

Detailed methods from this trial have been previously published [60]. Briefly, this study was a randomized controlled trial assessing the effects of a 4-month supervised aerobic and resistance exercise training compared to usual care. The primary outcome was metabolic syndrome, and the FRS was assessed as a secondary analysis at baseline and 4 months. Participants provided written informed consent. The University of Southern California Institutional Review Board approved the study. We enrolled breast cancer survivors who were: < 6 months out of treatment, non-smokers, inactive (<60 min/week of exercise), overweight, or obese (BMI ≥25kg/m^2^ or fat mass >30%, and a waist circumference >88cm). Race and ethnicity were self-reported. Participants were randomized to exercise or usual care: the exercise group performed a supervised one-on-one exercise program 3 days per week for 4 months. During the first and the third session of each week, women performed resistance (60–80% of 1-repetition maximum) and aerobic exercise (65–80% of maximum heart rate), and only aerobic exercise was done for the second session of the week. The FRS and the 10-year risk of cardiovascular disease were calculated using validated methods for women [57]. The 6 categories of the FRS were assessed: age, systolic blood pressure, LDL, HDL, presence of diabetes (glucose ≥ 100 mg/dL or taking diabetes medication), and smoking status. Age and smoking status were assessed at baseline. Resting blood pressure was assessed using an automated sphygmomanometer (Welch Allyn). HDL and LDL were analyzed in serum from fasting blood samples. Within-group differences in mean change for individual outcomes measured at 16 weeks were evaluated using general linear models repeated-measures analyses of variance. Between-group differences were evaluated with a mixed-model repeated-measures analysis. A priori covariates included age, type of treatment (i.e., chemotherapy, radiation therapy or both), surgery, medication use, BMI, and caloric intake.

Fifty-six Hispanic and Latina breast cancer survivors were included for the present secondary analyses. These women were randomized to exercise (*n*=29) or usual care (*n*=27) groups, mean age was 46±10 years, obese (34.9±6.2 kg/m^2^), and a similar percentage of pre and postmenopausal women. The adherence to the exercise intervention was 96%. Post-intervention total FRS was significantly reduced in the exercise group compared to the usual care group (−12.5; 95% CI −16.0, −4.0; *P* < 0.001), which corresponds to a 15% (95% CI: −18.0, −3.0; *P* < 0.001) decrease in the 10-year risk of developing cardiovascular disease (Table 2).

**Table 2:** Mean differences between exercise and usual care groups on Framingham risk score variables

Variable	Baseline	Post-intervention	Between group differences	
	Mean (SD)	Mean (SD)	Mean (95% CI)	*P* value†
Systolic blood pressure (mmHg)				
Exercise	140.1 (11.2)	119.9 (9.0)*	−16.9 (−20.2, −11.5)	<0.001
Usual care	138.9 (11.6)	138.0 (10.4)		
FRS pre-set point§				
Exercise	2.0 (1.0)	−3.0 (2.0)*	−3.0 (−5.0, −1.0)	0.001
Usual care	0.0 (2.0)	0.0 (2.0)		
HDL-C (mg/dL)				
Exercise	38.7 (7.1)	63.7 (7.7)*	27.2 (38.8, 15.3)	<0.001
Usual care	37.9 (6.8)	35.7 (7.1)		
FRS pre-set point§				
Exercise	2.0 (1.0)	−2.0 (1.5)*	4.0 (0.6, 6.3)	<0.001
Usual care	2.0 (1.0)	2.0 (2.0)		
LDL-C (mg/dL)				
Exercise	197.9 (21.7)	121.3 (22.1)*	−69.6 (−96.2, −17.6)	<0.001
Usual care	185.4 (19.3)	190.3 (19.7)		
FRS pre-set point§				
Exercise	2.0 (1.0)	0.0 (1.0)*	−2.0 (−4.5, −0.5)	0.001
Usual care	2.0 (1.0)	2.0 (2.0)		
Diagnosis of diabetes, *n* (%)				
Exercise	20 (69)	10 (34)*	−9.0 (−16.1, −5.4)	<0.001
Usual care	19 (70)	19 (70)		
FRS pre-set point§				
Exercise	2.0 (1.5)	1.0 (0.5)*	−1.0 (−2.5, −0.5)	0.003
Usual care	2.0 (1.0)	3.0 (1.0)		
Total FRS				
Exercise	12.0 (1.5)	2.0 (1.5)*	−12.5 (−16.0, −4.0)	<0.001
Usual care	12.0 (2.0)	13.0 (3.0)		
10-year risk (%)				
Exercise	13.0 (3.0)	1.0 (0.5)	−15.0 (−18.0, −3.0)	<0.001
Usual care	13.0 (3.0)	13.0 (3.0)		

*P value for repeated-measures ANOVA comparing changes in the exercise group from baseline to post-intervention, and in the usual care group from baseline to post-intervention

^†^*P* value for mixed model analysis comparing changes between the exercise and usual care group from baseline to post-intervention

^§^Assigned pre-set point for the respective variable based on calculating FRS to assess 10-yr CVD risk

Our results showed that a 16-week aerobic and resistance exercise intervention reduced the FRS and the 10-year risk of developing cardiovascular disease in Hispanic and Latina breast cancer survivors with overweight or obesity by reducing systolic blood pressure, LDL, the presence of diabetes, and increasing HDL. To our knowledge, this is the first report that explored the effects of exercise on the FRS in Hispanic and Latina breast cancer survivors. Our results showed exercise induced a 12.5 point reduction on the FRS and a 15% reduction in the predicted 10-year risk of developing cardiovascular disease, among Hispanic and Latina women, which is higher than the 9.5 points reduction on FRS and the 11% reduction found in our previous report including racially/ethnically diverse breast cancer survivors [58]. This larger exercise-induced effect on reducing cardiovascular disease risk among Hispanic and Latina women could be explained by higher prevalence of diabetes and lower physical activity levels at baseline in the Hispanic and Latina subsample at baseline compared with the full sample [58]. As such this may produce a stronger response to exercise as they are starting from a more deconditioned state, therefore greater room for improvement [61].

Strengths of the study include the recruitment of an ethnically diverse sample that allowed us to explore the effects of exercise on cardiovascular risk in this unrepresented population. As a limitation it is important to note that these are secondary analysis from a larger clinical trial, and therefore the study was not powered to assess cardiovascular disease risk. Also, the estimated 10-year risk reduction of developing cardiovascular disease after a 4-month intervention needs to be interpreted with caution since the sustainability of this exercise intervention over 10 years is unknown. Future large clinical trials that specifically target Hispanic and Latina breast cancer survivors are needed to corroborate our findings and to fully understand the potential of exercise to attenuate health disparities across breast cancer survivors.

Therefore, we found that a supervised aerobic and resistance exercise intervention reduces the 10-year risk of developing cardiovascular disease risk by 15%.

## Future Directions: Need for Culturally Tailored Exercise Interventions for Hispanic and Latina Breast Cancer Survivors to Target Cardiovascular Risk

The results of our exploratory study, as well as the review conducted, highlight the need of exercise interventions targeting cardiovascular risk factors in Hispanic and Latina breast cancer survivors. To bridge this gap in the cardio-oncology literature it is imperative to develop culturally tailored exercise interventions for this population. Culturally tailored interventions refer to the adaptations in the design, methods, and materials, as well as other components with the purpose of adapting the intervention to the cultural needs of a specific population [62].

Cultural considerations to tailor a cardio-oncology exercise intervention in Hispanic and Latina breast cancer survivors should aim to reduce first the specific structural and personal aforementioned barriers to exercise and research participation faced by this population. Components that should be considered include surface-structure components such as study materials translated to Spanish and bilingual trainers and staff [63]. Deep-structure components include the importance of incorporating Latina/o cultural values to create a more culturally sensitive program. Specifically, to integrate the values of familisimo (strong identification with and attachment to family), respeto (respect), confianza (trust), and simpatía (warmth, friendliness) [64]. Examples of how to incorporate these components include setting orientation sessions before the exercise intervention to build trust, conduct cultural sensitivity training for all staff involved in the trial, group sessions to facilitate community-building between participants, invite family members to participate in the sessions at the beginning of the trial to build social support, facilitate transportation, and include compensation for participation.

Furthermore, to reduce cardiovascular risk factors, a culturally tailored cardio-oncology exercise intervention should emphasize behavioral strategies to increase maintenance of exercise participation. In this regard, culturally tailored cardio-oncology lifestyle interventions should also incorporate theoretical frameworks to achieve behavior change. The Social Cognitive Theory (SCT) is commonly used to guide exercise interventions for breast cancer survivors [65, 66], and noted as the most frequently utilized theory in culturally sensitive nutrition and exercise interventions for Hispanic and Latina population [67]. This theoretical framework considers the influence of an individual’s experience, behavior, and environment on individual health behaviors [68]. Constructs of the SCT that can be incorporated when designing a culturally tailored cardio-oncology exercise intervention include environment, behavioral capability, expectations, self-control and performance, observational learning, and reinforcement (internal and external) [68]. Important aspects to emphasize maintenance of exercise participation includes goal setting, problem-solving barriers to participate in and access exercise resources, increasing social support while adopting exercise post-clinical intervention, self-monitoring of exercise performed, and rewarding oneself for meeting exercise goals.

Our laboratory is currently conducting two trials with cardiovascular risk factors outcomes that are culturally tailored to Hispanic and Latina breast cancer patients where we are using the strategies mentioned above [69, 70]. In both trials, we are using one-on-one home-based exercise virtually supervised via Zoom. This new home-based virtual setting could potentially help overcome barriers to exercise in this population including reducing travel burden and cost, time constraints, reaching participants living in rural areas, while maintaining the rigour of in-clinic training [71–73]. Our preliminary results of these trials show feasibility and great attendance data (results not published yet). Testing appropriate home-based exercise intervention strategies is increasingly important to offset cardio-oncology disparities in Hispanic and Latina women with breast cancer.

## Conclusions

In conclusion, most randomized exercise trials exploring cardiovascular risk factors for women with breast cancer have been composed primarily of highly educated White adults with mid-high socioeconomic status (SES) [74]. We have presented here the current evidence on exercise interventions measuring cardiovascular-related outcomes in Hispanic and Latina breast cancer survivors showing the clear need for well-powered and culturally tailored randomized controlled trials that evaluate cardiovascular risk in this population. Furthermore, we have presented our ethnicity-focused exploratory results from our randomized controlled trial, where we found that after 16 weeks of supervised aerobic and resistance exercise, overweight and obese Hispanic and Latina breast cancer survivors may reduce the 10-year risk of developing cardiovascular disease risk by 15%. This report highlights the importance of recruiting and retaining underrepresented minorities and the need to consider ethnicity when designing an exercise cardio-oncology clinical trial. Steps towards the development of culturally tailored cardio-oncology exercise interventions and increasing the lack of scientific data in this field for Hispanic and Latina women with breast cancer are needed in order to reduce cardio-oncology disparities.

## Data Availability

Data is available on request.
